# Domestic

**Published:** 1840-11-21

**Authors:** 


### DOMESTIC.

### CASES OF TETANUS TREATED SUCCESSFULLY.

*Case of Tetanus cured by the use of assafœtida, brandy, and tincture of rhubarb.*—Joseph Hutchinson, of New Hampshire, aged thirty-nine, a remarkably athletic man, employed as fireman on board of one of the Providence steamers, was admitted with symptoms of tetanus on the 16th of June.

About two weeks previous he had abraded the skin on the side of his left leg, over the cicatrix of an old ulcer. Subsequent to this accident he had been much exposed to sudden changes of temperature; his berth was leaky, and his clothes had been frequently wet. About a week before admission he had injured one of his fingers, which was still sore, but he thinks the stiffness about his throat had commenced before this latter accident. He continued at work until the 12th June. On Saturday, June 13th, he complained of pain in his back and of weakness. On the next day he had cramps in different parts of his body, extending particularly from his back along the region of the diaphragm, and afterwards to the lower extremities. He now sent for a physician who bled him, and gave him some cathartic medicine, which operated freely during the following day.

When admitted, his jaws were firmly closed, but not so completely as to prevent him from protruding his tongue slightly beyond his teeth; the lower jaw was somewhat retracted: he had frequent spasmodic twitchings in his body and limbs; his skin was bathed in profuse perspiration. The spasms were most severe in the left leg, the patella appeared to quiver beneath the hand when placed over it. His abdominal muscles were rigid, his pulse was ninety, his intellectual faculties undisturbed; his principal suffering was from the frequent recurrence of the cramps. The ulcer on his leg, produced by the abrasion, was in an irritable condition, and apparently spreading. ℞. Tincture assafœtida ℥ij. to be administered as an enema, and repeated every second hour. The ulcer was dressed with an anodyne and emollient poultice. Diet not restricted.

*June* 17. The patient had slept during the night, but never so long at a time as to require any intermission of the medicine. General symptoms as yesterday; spasms recurring every ten or fifteen minutes. In the afternoon they became excessively severe, throwing him into general convulsions. He was placed upon a bed on the floor to prevent him from falling. The enemata were now repeated at intervals of an hour, and the patient was further allowed a table-spoonful of brandy every second hour.

*June* 19. Spasms less severe, and recurring about once an hour, invariably coming on when he is about to fall asleep, and thus disturbing his repose. The enemata keep his bowels freely opened. He complains of thirst, and of a parched and slimy state of his mouth, for which he was directed porter diluted with water, to be taken ad libitum.

*June* 22. Spasms gradually growing less severe; pulse varying from one hundred to one hundred and ten; patient has had some repose at night, and has taken mild nourishment. The enemata have produced much soreness in the rectum and about the anus, in consequence of which they were stopped, and pills of gum assafœtida, each containing three grains, were given by the mouth; one pill every hour; the brandy and porter were continued as usual.

*June* 25. Spasms less and less frequent and severe. The patient's bowels have not been freely moved since the enemata were suspended. Stop the brandy, and take tincture of rhubarb as a substitute, in doses of half an ounce every two hours.

*June* 26. Scarcely any spasms since yesterday: pulse ninety; the rhubarb has acted gently upon the bowels.

*July* 6. Although he has had few or no spasms since last report, and has been able to sleep and to take regular nourishment of soft food, still the tonic rigidity of the muscles continues in most parts of the body. The patient is unable to move his legs, and passive motion gives him pain. To-day, for the first, the stiffness of the jaws suddenly left him: he felt, according to his own account, as if something had given way there. The ulcer in his leg had been for some time improving under simple dressings, and was now nearly healed.

Subsequent to this date, the rigidity of the back and limbs gradually yielded; the gastrocnemii muscles of the left leg, however, still continued rigid, and kept the foot permanently extended; but these, at length, by insensible degrees relaxed, and the patient regained the use of his legs. About the 15th of July he began to sit up; his limbs, however, were still remarkably feeble; he was at first obliged to use a crutch in walking, and suffered severely in attempting to flex his joints. After this date his recovery was rapid: but he did not leave the hospital until able to resume his work, on the 19th of August.

The other successful case of tetanus was in some respects peculiar; and though it occurred in private practice, I am induced to report it here, in connection with the preceding. I cannot describe it more accurately at present, than by simply transcribing the notes that were taken upon it at the time of its occurrence. I may premise, however, that it was in its early stage looked upon as an approaching attack of scarlatina; this disease being at the time prevalent.

*Case of Tetanus treated successfully.*—Nathaniel Scanlin, aged ten, a boy of plethoric habit, was taken ill Feb. 15th, 1838, with pain in his head, sore throat, pain in his bowels, and in the small of his back. He left school, unwell, in the morning; and was unable to eat his dinner. His mother, supposing him about to have an attack of croup, gave him an emetic, which operated, and followed this with a dose of castor oil, which had not yet operated when I saw him, at 7 o'clock, P. M. I then found him with flushed skin, eyes rather heavy; tongue discoloured with liquorice, but showing slight elevation of a few of the papillæ. The sublingual glands were inflamed, swollen, and painful: the fauces red, no apthæ upon them. The patient complained of soreness of the throat; he was too feeble to walk about, but was active enough in describing his symptoms, and sat up whilst I examined him. I simply directed warm catnip tea.

*February* 16. About an hour after my first visit, he was seized with convulsions, which lasted all night. Mustard draughts had been applied without relief; another physician, who was called to him early in the morning, administered three grains of calomel and an ordinary enema. When I saw him at noon, the convulsions were still upon him, recurring every fifteen or twenty minutes, passing off very suddenly and leaving him with general twitching, *like the twitching of tetanus.* His pulse was about one hundred, intermitting three or four times in a minute, and very weak. His skin was rather below the natural temperature. During the convulsions he did not froth at the mouth, and his respirations was performed by short, quick, and convulsive gasping. ℞. Tinct. Assafœtidæ ℥ss. to be mixed with an ounce of milk, administered as an injection, and to be repeated every second hour, while the fits continued. A warm bath was also given after the first enema.

5 *o'clock, P. M.* The enema had produced an immediate action of the bowels, and after its operation the child remained quiet for a longer time than at any former period since the commencement of the convulsions. Skin not above the natural temperature; tongue pasty and dark in the middle; pulse regular, weak, and at one hundred and two. The injections were continued, and the patient directed warm wine and water, made weak, sweetened, and to be taken as an occasional drink.

9 *o'clock, P. M.* Child sleeping soundly, pulse regular at one hundred; has taken his wine and water but once, and has not had a paroxysm within half an hour. During the intervals between the paroxysms, throughout the day, he has been rational; towards night he appeared to his friends to be at times delirious.

*February* 17, (in the morning at 9 o'clock.) The patient has slept well during the night; there have been three returns of the paroxysms within two hours; bowels moved three times since last visit, the evacuations being unhealthy and "like glue." Tongue pasty, and, over a red surface, coated with a thin white fur.

5 *o'clock, P. M.* Frequent returns of paroxysms, sometimes attended with swooning, which lasts a minute or more, sometimes characterized by twitching of the limbs, and sometimes by convulsions of the whole body. Whilst I was examining him, he suddenly clenched his fists, opened his mouth, stretched backwards, and had a severe general convulsion, with the spasmodic and rapid breathing noticed yesterday. During the fit his mouth remained open very wide, and his tongue retracted. Once or twice he bent forward as if to snap at the persons who were holding him in bed. The paroxysm lasted about two minutes, and then, suddenly leaving him, he sat up in bed, called for a drink, said his head was "all working," desired to have a bandage tied about it, and appeared as clear as ever. During the fit his pulse did not intermit; it had been all day about ninety-six. He had a slight cough, and complained of pain in his side.

Although the symptoms have hitherto been of equivocal character, yet this morning supposing them to bear much analogy to those of tetanus, I questioned with that view; and found on the last joint of the right thumb, a small bruise, about two lines broad, and covered with a dry scab. He stated that this had been caused by striking his knuckles against a rough projecting point of the stove pipe, on the 9th inst., just six days before he became unwell. The wound, he added, hurt him shockingly at first, and pained him very much all next day, but he had thought nothing of it afterwards.

*February* 18. Slept tolerably during the night; four convulsions in the course of the day; throat not so sore as formerly; tongue clearing off; skin natural, pulse eighty-four, bowels not moved.

℞. Ol. Ricini ℥j.; and if the fits return, resort to former treatment.

*February* 19. During the day better than usual, having had but three or four mild returns of the paroxysms. At night sitting up in bed, free from pain, playing with one of his little companions. Oil had operated freely, tongue quite clean, appetite returning. The symptoms of the case are certainly not those usually attending tetanus, yet this is doubtless the disease affecting him.

*February* 20. In the morning he had another convulsion, which lasted four or five minutes, and left him with some confusion of intellect, and slight injection of the eyes. He had a few drops of blood discharged from the nostrils. Bowels not moved since yesterday. Repeat the castor oil. At the evening visit I found that the oil had vomited him, and that he had had two slight paroxysms since the morning. Have sulph. magnes. ℥ss.

*February* 21. No return of paroxysms: the cathartic has produced healthy evacuations; patient apparently well.

*February* 22. No further returns of paroxysms. Discharged cured.

*Remarks.*—I have neglected to note in this case the date at which the assafœtida was suspended. Its effects were more decided on the first day than at any time subsequently, and it was entirely neglected towards the last.

As I had no knowledge of this article ever having before been employed as a remedy for tetanus, I was induced, by the successful result of this case, to try it again at the next favourable opportunity. The second patient to whom I administered it, was a lad aged fourteen, who had received a severe gun-shot wound, involving the right groin, right testicle, and upper part of the right thigh. The wound, which had been caused by the accidental discharge of a gun on the 25th of March, 1838, was of itself sufficient to have destroyed life. Symptoms of tetanus ensued, on the morning of the 31st of March, between five and six days from the date of injury; and the patient died on the day following. Owing to the state of the wound, the tincture of assfœtida could not be administered by the rectum. It was therefore given by the mouth; and was not carefully continued, the patient refusing it on account of the difficulty of swallowing.

The third instance in which this remedy was administered, was the fatal case referred to in a former part of this report. The disease had existed several days, and the patient died of suffocation in attempting to swallow the first dose of the tincture, which was unfortunately administered by the mouth. The fourth case was that of Hutchinson, who recovered. It has since been employed in the hospital in another case, a female, a patient of Dr. Post, but without success.

Thus, out of five cases in which assafœtida has been used, two have terminated favourably; but how far this success is attributable to the treatment, is as yet impossible to determine. I have no doubt that many patients, suffering under this formidable malady, are over-treated; yet we are not in the habit of hearing of spontaneous cures; nor, indeed, of any great proportion of successful cases, even under the most judicious management. It is to be feared that no course of treatment can ever prove generally successful; hence any remedy that is attended even with occasional success, is worthy of the notice of the profession.

In resorting to the assafœtida again, I should prefer administering it by the rectum; but if this plan be inadmissible, it must be given by the mouth. Under these circumstances, the gum is preferable to the tincture, being less apt to excite spasmodic action of the muscles of the throat, and thus lead to suffocation.—*Dr. Watson's Hospital Reports in New York Journal of Medicine and Surgery.*

